# High-quality genome assembly and annotation of inactivated animal vaccine bacteria strains in South Korea

**DOI:** 10.1186/s12863-026-01475-x

**Published:** 2026-08-05

**Authors:** Yeonkyeong Lee, Jin-Ju Nah, Su-Min Go, Hyun-Ok Ku, Il Jang

**Affiliations:** Veterinary Drugs and Biologics Division, Animal and Plant Quarantine Agency, 177, Hyeoksin 8-ro, Gimcheon-si, Gyeongsangbuk-do Korea

**Keywords:** Hybrid assembly, Inactivated vaccine, Seed lot system, *Pasteurella multocida*, *Actinobacillus pleuropneumoniae*, *Glaesserella parasuis*, *Mannheimia haemolytica*, *Avibacterium paragallinarum*, *Escherichia coli*

## Abstract

**Objectives:**

Production of high-quality biological products depends on the careful selection and consistent management of seed strains. In this study, we generated complete genome sequences of bacterial strains employed in inactivated animal vaccines in South Korea and comprehensively examined their gene annotations. The genome data thus obtained provide a solid basis for confirming, at the strain level, whether manufacturers are using the same seed strains over time. In addition, these data offer a useful reference resource to support stable large-scale production and quality control of biological products.

**Data description:**

The complete genomes of eleven bacterial strains employed for inactivated vaccine production were generated and annotated. A hybrid assembly workflow combining Illumina short reads (NovaSeq 6000) with Oxford Nanopore long reads (MinION) produced high-quality, gap-free genomes for all strains, with genome sizes ranging from 2.28 Mb to 5.35 Mb. The strains comprised *Pasteurella multocida* (D, 3A, A), *Actinobacillus pleuropneumoniae* (2 and 5), *Glaesserella parasuis* 4, *Mannheimia haemolytica* KO, *Avibacterium paragallinarum* C, and three *Escherichia coli* strains (F41, YC21-F17, K99S). All assemblies exhibited high completeness (>99%) and minimal contamination (<1%), ensuring reliable downstream genomic characterization.

## Objective

A fundamental principle in the manufacturing of high-quality biological products is the selection and systematic control of appropriate seed strains. Following the selection of a suitable strain, a single viable unit is expanded to generate a master seed lot, and working seed lots are then produced from this master lot for vaccine manufacturing. In a seed lot system, each vaccine batch is derived from the same master seed lot at a defined passage level. This approach minimizes variation that could arise from repeated subculturing [1, 2]. As part of this system, identity testing is conducted to verify that the strains present in the biological products are identical to the originally registered seed strains.

Whole-genome analysis has recently become a widely used method for confirming the identity of vaccine strains [3–5]. In this study, we conducted whole-genome sequencing and analysis of bacterial strains used in inactivated vaccines licensed for veterinary use. The study aimed to generate reference genome data that can support future strain identification and quality control of these vaccine strains. Although the complete genome sequences were deposited and made publicly available with permission from the manufacturers, the commercial product names are not disclosed because this was not authorized.

### Data description

Eleven bacterial strains were analyzed using whole-genome sequencing:

*Pasteurella multocida* D, 3A, and A;

*Actinobacillus pleuropneumoniae* 2 and 5;

*Glaesserella parasuis* 4;

*Mannheimia haemolytica* KO;

*Avibacterium paragallinarum* C;

and *Escherichia coli* F41, YC21-F17, and K99S.

The designators used in this manuscript (D, 3A, A, 2, 5, 4, KO, C, F41, YC21-F17, and K99S) are the actual seed/strain identifiers recognized by the respective manufacturers.

All strains were obtained as seed stocks used for each inactivated vaccine, provided by the manufacturers. To obtain high-molecular-weight DNA suitable for both short- and long-read sequencing, nucleic acids were extracted from each bacterial stock using a Maelstrom 4810 automated nucleic acid extraction system (TANBead, Taoyuan City, Taiwan) with the Nucleic Acid Extraction Kit, following the manufacturer’s instructions. The extracted DNA was further purified using AMPure XP beads (Beckman Coulter, Brea, CA, USA) to remove residual impurities and short DNA fragments. DNA purity was assessed using a NanoDrop spectrophotometer, and DNA quantity was measured using a Qubit fluorometer (Thermo Fisher Scientific, Waltham, MA, USA) prior to library preparation.

To obtain complete and high-quality assemblies, we used a hybrid genome assembly technique that combined short- and long-read data. For short-read sequencing, libraries were prepared with the NEXTflex^®^ Rapid DNA-Seq Kit V2 and NEXTflex^®^ Unique Dual Index Barcodes (Revvity, Waltham, MA, USA), followed by sequencing as 150-bp paired-end reads on an Illumina NovaSeq 6000 instrument (Illumina, San Diego, CA, USA). For long-read sequencing, libraries were generated using the SQK-NBD114.24 Native Barcoding Kit 24 V14 (Oxford Nanopore Technologies, Oxford, UK) with the NEBNext^®^ FFPE DNA Repair Mix (New England Biolabs, Ipswich, MA, USA), following the manufacturer’s instructions. Sequencing was performed on an ONT MinION device with a FLO-MIN114 flow cell (R10.4.1; Oxford Nanopore Technologies) for more than 72 h. Long-read libraries were multiplexed on MinION flow cells using the Native Barcoding Kit, and the resulting per-sample read counts and sequencing depth are provided in Data file 1 [6]. Raw nanopore reads (POD5) were base-called using Dorado (version 1.1.1) with the super-accuracy model (dna_r10.4.1_e8.2_400bps_sup@v5.0.0) to generate FASTQ files.

Summary statistics of the raw sequencing data are presented in Data file 1 [6], and the raw reads have been deposited in the NCBI Sequence Read Archive (Table 1, Data set 1) [7]. For subsequent analysis, the Illumina reads were trimmed using Trimmomatic (v0.39) [8] to eliminate adapter sequences and low-quality bases.

Nanopore reads were quality-filtered using Filtlong (v0.2.1) [9] with a minimum read length of 1,500 bp. Long-read assembly was performed using Autocycler (v0.6.2) [10]. Only circularized contigs were retained for downstream analysis. The resulting assemblies were polished sequentially using Medaka (v2.2.1) [11] with the r1041_e82_400bps_sup_v5.2.0 model for long-read error correction, followed by Polypolish (v0.6.0) [12] for short-read polishing. For Polypolish, Illumina reads were independently aligned to the assembly using BWA-MEM (v0.7.17) [13] and filtered using the polypolish filter function prior to polishing. Plasmid sequences were identified and quality-controlled using MOB-recon (MOB-suite v3.1.9) [14]. Contigs other than the primary chromosome that were classified as chromosomal by MOB-recon but carrying relaxase or replicon sequences, or exceeding 1,000 bp, were further verified by BLASTn against the NCBI nucleotide (nt) database. Contigs showing ≥95% nucleotide identity and ≥80% query coverage to known plasmid sequences were reclassified as plasmids. Contigs matching host genomic sequences or lacking significant database hits were excluded.

We evaluated the quality of the assembly (number of contigs, total length, N50, and GC content) with QUAST (v5.2.0) [15]. Gene prediction and annotation were carried out using the NCBI Prokaryotic Genome Annotation Pipeline (PGAP) [16]. Complete circular genomes were visualized with CGView [17], and CheckM (version 1.2.3) [18] was applied to estimate genome completeness and contamination based on lineage-specific single-copy marker genes.

We obtained complete, high-quality single-circular chromosomes for all 11 bacterial vaccine strains. For a subset of strains, circular plasmid sequences were also resolved. All complete genome assemblies have been deposited in GenBank, and individual nucleotide accession numbers have been assigned to each chromosome and plasmid; these are listed, together with the corresponding genome assembly accessions, in Table 1 (Data sets 2–36) [19–53]. The main genomic features of each vaccine strain are summarized in Data files 2, 3, and 4 (Table 1) [6].

The genome of *Pasteurella multocida* D consisted of a circular chromosome (CM135142, 2,423,566 bp, 40.16% GC) and a circular plasmid (JBSQFY020000002, 2,613 bp, 38.08% GC). The complete genome assembly is available under GenBank assembly accession GCA_054171665. The assembly showed 100% completeness, 0.38% contamination, 2,256 protein-coding genes, 58 tRNAs, 19 rRNAs, and 4 ncRNAs (Table 1, Data file 5, Data sets 2–4) [6, 19–21].

The genome of *Pasteurella multocida* 3A consisted of a circular chromosome (CM135141, 2,289,143 bp, 40.39% GC) and a circular plasmid (JBSQFZ020000002, 4,612 bp, 41.50% GC). The complete genome assembly is available under GenBank assembly accession GCA_054171645. The assembly showed 100% completeness, 0.12% contamination, 2,056 protein-coding genes, 56 tRNAs, 19 rRNAs, and 4 ncRNAs (Table 1, Data file 6, Data sets 5–7) [6, 22–24].

The genome of Actinobacillus pleuropneumoniae 2 consisted of a single circular chromosome (CM135140, 2,282,705 bp, 41.19% GC). The complete genome assembly is available under GenBank assembly accession GCA_054171625. The assembly showed 100% completeness, 0.04% contamination, 2,046 protein-coding genes, 60 tRNAs, 20 rRNAs, and 4 ncRNAs (Table 1, Data file 7, Data sets 8 and 9) [6, 25, 26].

The genome of *Actinobacillus pleuropneumoniae* 5 consisted of a circular chromosome (CM135139, 2,300,843 bp, 41.28% GC) and a circular plasmid (JBSQGB020000002, 7,694 bp, 60.90% GC). The complete genome assembly is available under GenBank assembly accession GCA_054171605. The assembly showed 100% completeness, 0.18% contamination, 2,064 protein-coding genes, 61 tRNAs, 19 rRNAs, and 4 ncRNAs (Table 1, Data file 8, Data sets 10–12) [6, 27–29].

The genome of Glaesserella parasuis 4 consisted of a single circular chromosome (CM135138, 2,298,776 bp, 40.02% GC). The complete genome assembly is available under GenBank assembly accession GCA_054171585. The assembly showed 100% completeness, 0.84% contamination, 2,069 protein-coding genes, 59 tRNAs, 19 rRNAs, and 4 ncRNAs (Table 1, Data file 9, Data sets 13 and 14) [6, 30, 31].

The genome of Pasteurella multocida A consisted of a single circular chromosome (CM135137, 2,373,932 bp, 40.29% GC). The complete genome assembly is available under GenBank assembly accession GCA_054171565. The assembly showed 100% completeness, 0.14% contamination, 2,172 protein-coding genes, 58 tRNAs, 19 rRNAs, and 4 ncRNAs (Table 1, Data file 10, Data sets 15 and 16) [6, 32, 33].

The genome of Mannheimia haemolytica KO consisted of a single circular chromosome (CM135136, 2,694,182 bp, 41.03% GC). The complete genome assembly is available under GenBank assembly accession GCA_054171545. The assembly showed 100% completeness, 0.15% contamination, 2,625 protein-coding genes, 66 tRNAs, 20 rRNAs, and 4 ncRNAs (Table 1, Data file 11, Data sets 17 and 18) [6, 34, 35].

The genome of Avibacterium paragallinarum C consisted of a single circular chromosome (CM135135, 2,689,475 bp, 41.19% GC). The complete genome assembly is available under GenBank assembly accession GCA_054171525. The assembly showed 100% completeness, 0.09% contamination, 2,644 protein-coding genes, 60 tRNAs, 19 rRNAs, and 4 ncRNAs (Table 1, Data file 12, Data sets 19 and 20) [6, 36, 37].

The genome of *Escherichia coli* F41 consisted of a circular chromosome (CM135134, 4,887,336 bp, 50.73% GC) and four circular plasmids (JBSQGG020000002–JBSQGG020000005, sizes ranging from 3,555 bp to 113,783 bp). The complete genome assembly is available under GenBank assembly accession GCA_054171505. The assembly showed 100% completeness, 0.05% contamination, 4,682 protein-coding genes, 88 tRNAs, 22 rRNAs, and 13 ncRNAs (Table 1, Data file 13, Data sets 21–26) [6, 38–43].

The genome of *Escherichia coli* YC21-F17 consisted of a circular chromosome (CM176886, 5,218,516 bp, 50.77% GC) and three circular plasmids (JBYUJG010000002-JBYUJG010000004, sizes ranging from 6,079 bp to 127,324 bp). The complete genome assembly is available under GenBank assembly accession GCA_058136955. The assembly showed 100% completeness, 0.28% contamination, 4,931 protein-coding genes, 92 tRNAs, 22 rRNAs, and 10 ncRNAs (Table 1, Data file 14, Data sets 27–31) [6, 44–48].

The genome of *Escherichia coli* K99S consisted of a circular chromosome (CM135132, 4,824,770 bp, 50.77% GC) and three circular plasmids (JBSQGI020000002–JBSQGI020000004, sizes ranging from 3,555 bp to 110,777 bp). The complete genome assembly is available under GenBank assembly accession GCA_054171465. The assembly showed 100% completeness, 0.14% contamination, 4,516 protein-coding genes, 88 tRNAs, 22 rRNAs, and 13 ncRNAs (Table 1, Data file 15, Data sets 32–36) [6, 49–53].

**Table 1:** Overview of data files/data sets

Label	Name of data file/data set	File types (file extension)	Data repository and identifier (DOI or accession number)
Data file 1	Raw data (Illumina and MinION) details	MS Excel file (.xlsx)	Figshare: 10.6084/m9.figshare.30876641 [6]
Data file 2	Statistics of de novo assembly	MS Excel file (.xlsx)	Figshare: 10.6084/m9.figshare.30876641 [6]
Data file 3	Detailed features of chromosomes	MS Excel file (.xlsx)	Figshare: 10.6084/m9.figshare.30876641 [6]
Data file 4	Detailed features of plasmids	MS Excel file (.xlsx)	Figshare: 10.6084/m9.figshare.30876641 [6]
Data file 5	Circular map of *Pasteurella multocida* D	PDF (.pdf)	Figshare: 10.6084/m9.figshare.30876641 [6]
Data file 6	Circular map of *Pasteurella multocida* 3 A	PDF (.pdf)	Figshare: 10.6084/m9.figshare.30876641 [6]
Data file 7	Circular map of *Actinobacillus pleuropneumoniae* 2	PDF (.pdf)	Figshare: 10.6084/m9.figshare.30876641 [6]
Data file 8	Circular map of *Actinobacillus pleuropneumoniae* 5	PDF (.pdf)	Figshare: 10.6084/m9.figshare.30876641 [6]
Data file 9	Circular map of *Glaesserella parasuis* 4	PDF (.pdf)	Figshare: 10.6084/m9.figshare.30876641 [6]
Data file 10	Circular map of *Pasteurella multocida* A	PDF (.pdf)	Figshare: 10.6084/m9.figshare.30876641 [6]
Data file 11	Circular map of *Mannheimia haemolytica* KO	PDF (.pdf)	Figshare: 10.6084/m9.figshare.30876641 [6]
Data file 12	Circular map of *Avibacterium paragallinarum* C	PDF (.pdf)	Figshare: 10.6084/m9.figshare.30876641 [6]
Data file 13	Circular map of *Escherichia coli* F41	PDF (.pdf)	Figshare: 10.6084/m9.figshare.30876641 [6]
Data file 14	Circular map of *Escherichia coli* YC21-F17	PDF (.pdf)	Figshare: 10.6084/m9.figshare.30876641 [6]
Data file 15	Circular map of *Escherichia coli* K99S	PDF (.pdf)	Figshare: 10.6084/m9.figshare.30876641 [6]
Data set 1	Raw sequencing data (Illumina and MinION) of eleven strains	FASTQ files (.fastq)	NCBI data: https://identifiers.org/bioproject:PRJNA1178821 [7]
Data set 2	Chromosome sequence of *Pasteurella multocida* D	Standard FASTA file (.fasta)	NCBI data: https://identifiers.org/insdc:CM135142 [19]
Data set 3	Plasmid pD_1 sequence of *Pasteurella multocida* D	Standard FASTA file (.fasta)	NCBI data: https://identifiers.org/ncbi/insdc:JBSQFY020000002 [20]
Data set 4	Genome assembly of *Pasteurella multocida* D	FASTA and GenBank flat files (.fasta, .gbff)	NCBI data: https://identifiers.org/insdc.gca:GCA_054171665 [21]
Data set 5	Chromosome sequence of *Pasteurella multocida* 3A	Standard FASTA file (.fasta)	NCBI data: https://identifiers.org/insdc:CM135141 [22]
Data set 6	Plasmid p3A_1 sequence of *Pasteurella multocida* 3A	Standard FASTA file (.fasta)	NCBI data: https://identifiers.org/ncbi/insdc:JBSQFZ020000002 [23]
Data set 7	Genome assembly of *Pasteurella multocida* 3A	FASTA and GenBank flat files (.fasta, .gbff)	NCBI data: https://identifiers.org/insdc.gca:GCA_054171645 [24]
Data set 8	Chromosome sequence of *Actinobacillus pleuropneumoniae* 2	Standard FASTA file (.fasta)	NCBI data: https://identifiers.org/insdc:CM135140 [25]
Data set 9	Genome assembly of *Actinobacillus pleuropneumoniae* 2	FASTA and GenBank flat files (.fasta, .gbff)	NCBI data: https://identifiers.org/insdc.gca:GCA_054171625 [26]
Data set 10	Chromosome sequence of *Actinobacillus pleuropneumoniae* 5	Standard FASTA file (.fasta)	NCBI data: https://identifiers.org/insdc:CM135139 [27]
Data set 11	Plasmid p5_1 sequence of *Actinobacillus pleuropneumoniae* 5	Standard FASTA file (.fasta)	NCBI data: https://identifiers.org/ncbi/insdc:JBSQGB020000002 [28]
Data set 12	Genome assembly of *Actinobacillus pleuropneumoniae* 5	FASTA and GenBank flat files (.fasta, .gbff)	NCBI data: https://identifiers.org/insdc.gca:GCA_054171605 [29]
Data set 13	Chromosome sequence of *Glaesserella parasuis* 4	Standard FASTA file (.fasta)	NCBI data: https://identifiers.org/insdc:CM135138 [30]
Data set 14	Genome assembly of *Glaesserella parasuis* 4	FASTA and GenBank flat files (.fasta, .gbff)	NCBI data: https://identifiers.org/insdc.gca:GCA_054171585 [31]
Data set 15	Chromosome sequence of *Pasteurella multocida* A	Standard FASTA file (.fasta)	NCBI data: https://identifiers.org/insdc:CM135137 [32]
Data set 16	Genome assembly of *Pasteurella multocida* A	FASTA and GenBank flat files (.fasta, .gbff)	NCBI data: https://identifiers.org/insdc.gca:GCA_054171565 [33]
Data set 17	Chromosome sequence of *Mannheimia haemolytica* KO	Standard FASTA file (.fasta)	NCBI data: https://identifiers.org/insdc:CM135136 [34]
Data set 18	Genome assembly of *Mannheimia haemolytica* KO	FASTA and GenBank flat files (.fasta, .gbff)	NCBI data: https://identifiers.org/insdc.gca:GCA_054171545 [35]
Data set 19	Chromosome sequence of *Avibacterium paragallinarum* C	Standard FASTA file (.fasta)	NCBI data: https://identifiers.org/insdc:CM135135 [36]
Data set 20	Genome assembly of *Avibacterium paragallinarum* C	FASTA and GenBank flat files (.fasta, .gbff)	NCBI data: https://identifiers.org/insdc.gca:GCA_054171525 [37]
Data set 21	Chromosome sequence of *Escherichia coli* F41	Standard FASTA file (.fasta)	NCBI data: https://identifiers.org/insdc:CM135134 [38]
Data set 22	Plasmid pF41_1 sequence of *Escherichia coli* F41	Standard FASTA file (.fasta)	NCBI data: https://identifiers.org/ncbi/insdc:JBSQGG020000002 [39]
Data set 23	Plasmid pF41_2 sequence of *Escherichia coli* F41	Standard FASTA file (.fasta)	NCBI data: https://identifiers.org/ncbi/insdc:JBSQGG020000003 [40]
Data set 24	Plasmid pF41_3 sequence of *Escherichia coli* F41	Standard FASTA file (.fasta)	NCBI data: https://identifiers.org/ncbi/insdc:JBSQGG020000004 [41]
Data set 25	Plasmid pF41_4 sequence of *Escherichia coli* F41	Standard FASTA file (.fasta)	NCBI data: https://identifiers.org/ncbi/insdc:JBSQGG020000005 [42]
Data set 26	Genome assembly of *Escherichia coli* F41	FASTA and GenBank flat files (.fasta, .gbff)	NCBI data: https://identifiers.org/insdc.gca:GCA_054171505 [43]
Data set 27	Chromosome sequence of *Escherichia coli* YC21-F17	Standard FASTA file (.fasta)	NCBI data: https://identifiers.org/insdc:CM176886 [44]
Data set 28	Plasmid pYC21-F17_1 sequence of *Escherichia coli* YC21-F17	Standard FASTA file (.fasta)	NCBI data: https://identifiers.org/ncbi/insdc:JBYUJG010000002 [45]
Data set 29	Plasmid pYC21-F17_2 sequence of *Escherichia coli* YC21-F17	Standard FASTA file (.fasta)	NCBI data: https://identifiers.org/ncbi/insdc:JBYUJG010000003 [46]
Data set 30	Plasmid pYC21-F17_3 sequence of *Escherichia coli* YC21-F17	Standard FASTA file (.fasta)	NCBI data: https://identifiers.org/ncbi/insdc:JBYUJG010000004 [47]
Data set 31	Genome assembly of *Escherichia coli* YC21-F17	FASTA and GenBank flat files (.fasta, .gbff)	NCBI data: https://identifiers.org/insdc.gca:GCA_058136955 [48]
Data set 32	Chromosome sequence of *Escherichia coli* K99S	Standard FASTA file (.fasta)	NCBI data: https://identifiers.org/insdc:CM135132 [49]
Data set 33	Plasmid pK99S_1 sequence of *Escherichia coli* K99S	Standard FASTA file (.fasta)	NCBI data: https://identifiers.org/ncbi/insdc:JBSQGI020000002 [50]
Data set 34	Plasmid pK99S_2 sequence of *Escherichia coli* K99S	Standard FASTA file (.fasta)	NCBI data: https://identifiers.org/ncbi/insdc:JBSQGI020000003 [51]
Data set 35	Plasmid pK99S_3 sequence of *Escherichia coli* K99S	Standard FASTA file (.fasta)	NCBI data: https://identifiers.org/ncbi/insdc:JBSQGI020000004 [52]
Data set 36	Genome assembly of *Escherichia coli* K99S	FASTA and GenBank flat files (.fasta, .gbff)	NCBI data: https://identifiers.org/insdc.gca:GCA_054171465 [53]

## Data Availability

All data records generated in this study are listed in Table 1 and are cited individually in the reference list [6, 7, 19–53]. The raw sequencing data (Illumina and MinION) generated and analyzed in the current study have been submitted to the NCBI Sequence Read Archive under BioProject accession number PRJNA1178821 (https://identifiers.org/bioproject:PRJNA1178821) (Data set 1) [7]. The BioSample numbers for each strain are SAMN53536558, SAMN53536559, SAMN53536560, SAMN53536561, SAMN53536562, SAMN53536563, SAMN53536564, SAMN53536565, SAMN53536566, SAMN53536568, and SAMN60560795 (https://identifiers.org/biosample:SAMN53536558 to https://identifiers.org/biosample:SAMN53536566, https://identifiers.org/biosample:SAMN53536568 and https://identifiers.org/biosample:SAMN60560795). The raw data details, de novo assembly statistics, chromosome and plasmid features, and circular genome maps of the eleven strains are available in the Figshare repository (10.6084/m9.figshare.30876641) (Data files 1–15) [6]. The chromosome sequences, plasmid sequences, and genome assemblies of the eleven strains have been deposited in the NCBI GenBank database and are provided in Table 1 with direct links to each record: Pasteurella multocida D (Data sets 2–4) [19–21], Pasteurella multocida 3A (Data sets 5–7) [22–24], Actinobacillus pleuropneumoniae 2 (Data sets 8 and 9) [25, 26], Actinobacillus pleuropneumoniae 5 (Data sets 10–12) [27–29], Glaesserella parasuis 4 (Data sets 13 and 14) [30, 31], Pasteurella multocida A (Data sets 15 and 16) [32, 33], Mannheimia haemolytica KO (Data sets 17 and 18) [34, 35], Avibacterium paragallinarum C (Data sets 19 and 20) [36, 37], Escherichia coli F41 (Data sets 21–26) [38–43], Escherichia coli YC21-F17 (Data sets 27–31) [44–48], and Escherichia coli K99S (Data sets 32–36) [49–53].
